# Editorial: Beer - from tradition to innovation

**DOI:** 10.3389/fnut.2024.1536519

**Published:** 2024-12-17

**Authors:** Claudia Gonzalez Viejo, Carmen Hernandez-Brenes, Sigfredo Fuentes

**Affiliations:** ^1^Digital Agriculture, Food and Wine Research Group, School of Agriculture, Food and Ecosystem Sciences. Faculty of Science, The University of Melbourne, Parkville, VIC, Australia; ^2^Tecnologico de Monterrey, School of Engineering and Science, Monterrey, NL, Mexico

**Keywords:** industry transformation, sustainability, optimized brewing, metabolomics, emerging technologies

## Introduction

Beer is the most consumed alcoholic beverage worldwide in terms of volume and is currently undergoing significant transformation. Consumer demand for healthier, higher-quality products is driving innovation in brewing, which now integrates traditional methods with cutting-edge technologies—from novel digital technologies such as computer vision, machine learning, and sensors to robotics and artificial intelligence (AI)—offering high-quality beer products that can be tailored to consumer demands. These technologies provide significant advantages, such as reduced time and costs in the beer production chain, from the field to the glass. This Research Topic contributes directly to this advancement by comprehensively investigating beer's multifaceted role in human health, evaluating contemporary innovations in brewing methods, and exploring the potential of emerging technologies. However, with the current emergence of AI-driven technologies, researchers should drive an industry transformation to integrate tradition with innovation, from the field to the glass. This could contribute to producing higher-quality beer that is more appealing and palatable, thereby meeting consumers' needs.

The research presented in the publications comprising this Research Topic addresses several key areas. Zhang et al. conducted a mini-review on the interaction between beer consumption, the gut microbiome, and immune system function. This review highlighted the presence of bioactive compounds in beer, particularly polyphenols and fiber, which significantly modulated the gut microbiome and influenced various aspects of the immune response. The findings suggested a potential link between moderate beer consumption and positive health effects, including anti-carcinogenic effects, improvements in cardiovascular events, and the management of metabolic syndrome. Cahuê et al. investigated the ergogenic and satiety effects of a non-alcoholic beer microparticle beverage on Wistar rats using a controlled exercise regime. They found a dose-dependent effect, with lower doses enhancing aerobic performance and higher doses promoting satiety. This study highlighted the potential of innovative beer formulations as both functional beverages and dietary supplements, particularly for athletes. The precise mechanisms underlying these effects, including potential interactions with the gut microbiome and the role of specific bioactive components, remain significant areas for future exploration.

On the other hand, Goodman-Smith et al. conducted a New Zealand-based case study evaluating consumer acceptance of upcycled craft beer that utilizes surplus bread in the brewing process. The authors used online surveys and in-store sampling, and their findings suggested that in-store experiences enhance consumer awareness and acceptance of upcycled foods. The study's findings offer valuable insights for developing effective marketing strategies for upcycled food products, thereby contributing to sustainability.

Pieczonka et al. proposed an innovative, high-resolution metabolomic analysis of the brewing process using ultra-high-resolution mass spectrometric analysis (FT-ICR-MS). Their study characterized the molecular changes occurring throughout beer production, identifying distinct molecular signatures associated with specific steps in the brewing process. This detailed, large-scale analysis offers an advancement in understanding the brewing process at a molecular level, providing a basis for refining brewing techniques, improving quality control, and potentially identifying novel bioactive compounds.

## Conclusion

The studies published under this Research Topic significantly advance the field of beer science by bridging the gap between traditional knowledge and innovative findings, contributing to both beer composition and health-related topics. The studies reveal a multifaceted relationship between beer, human health, and sustainable food practices. Future research can build on these findings to further explain the complex mechanisms involved, optimize brewing techniques, and fully realize the potential of beer in human health and nutrition. Furthermore, although large breweries are increasingly interested in implementing AI across their processes, from the barley to the final product, few researchers have developed AI technologies to ensure and assess beer quality at various production stages. Therefore, there is a need for further research and development of novel technologies, such as computer vision, robotics, sensors, machine learning modeling, and digital twins, to address current global trends and requirements, which will aid in offering better products to consumers.

